# Genome-Wide Identification of the Geranylgeranyl Pyrophosphate Synthase (GGPS) Gene Family Associated with Natural Rubber Synthesis in *Taraxacum kok-saghyz* L. Rodin

**DOI:** 10.3390/plants13192788

**Published:** 2024-10-04

**Authors:** Lili Wang, Huan He, Jiayin Wang, Zhuang Meng, Lei Wang, Xiang Jin, Jianhang Zhang, Pingping Du, Liyu Zhang, Fei Wang, Hongbin Li, Quanliang Xie

**Affiliations:** 1 Key Laboratory of Xinjiang Phytomedicine Resource and Utilization of Ministry of Education, Xinjiang Production and Construction Corps Key Laboratory of Oasis Town and Mountain-basin System Ecology, College of Life Sciences, Shihezi University, Shihezi 832003, China; lfyroquz@163.com (L.W.); he_huan026@163.com (H.H.); wjyinee@163.com (J.W.); zhuangmeng610@163.com (Z.M.); wangleibailu@163.com (L.W.); zjh17630412656@163.com (J.Z.); dopingping@126.com (P.D.); zhangliyu981125@163.com (L.Z.); 2 Ministry of Education Key Laboratory for Ecology of Tropical Islands, Key Laboratory of Tropical Animal and Plant Ecology of Hainan Province, College of Life Sciences, Hainan Normal University, Haikou 571158, China; jinx@hainnu.edu.cn

**Keywords:** geranylgeranyl pyrophosphate synthase, *Taraxacum kok-saghyz*, natural rubber, gene expression, latex, chloroplast, ethylene, methyl jasmonate

## Abstract

*Taraxacum kok-saghyz* Rodin (TKS) is a recognized alternative source of natural rubber comparable to the rubber tree. The geranylgeranyl pyrophosphate synthase (GGPS) catalyzed the synthesis of geranylgeranyl pyrophosphate (GGPP), which is an important enzyme in the secondary metabolism pathway. In this study, we present the first analysis of the *GGPS* gene family in TKS, where a total of seven *TkGGPS* family members were identified. Their core motifs, conserved structural domains, gene structures, and cis-acting elements were described. In addition, two phylogenetic trees were constructed based on the Neighbor-Joining and Maximum-Likelihood methods, and the *TkGGPSs* were highly conserved and exhibited good collinearity with the other species. Transcriptome data showed that seven *TkGGPS* gene members were expressed in all the 12 tissues measured, and *TkGGPS1*, *TkGGPS3*, and *TkGGPS6* were highly expressed in latex, suggesting that they may be associated with natural rubber synthesis. Meanwhile, quantitative real-time PCR (qRT-PCR) showed that the expression levels of the *TkGGPS* genes were regulated by the ethylene and methyl jasmonate (MeJA) pathways. Subcellular localization results indicated that all the TkGGPS proteins were also located in chloroplasts involved in photosynthesis in plants. This study will provide valuable insights into the selection of candidate genes for molecular breeding and natural rubber biosynthesis in TKS.

## 1. Introduction

Natural rubber (NR) is a kind of natural polymer compound with cis-1,4-polyisoprene as the main component [1,2]. More than 2500 plants in the world can produce natural rubber, but only a few of these species can produce high-molecular-weight rubber [3,4]. It is widely used in the fields of industry, agriculture, national defense, transportation, medicine, and daily life [5,6,7] because of its high strength, toughness, elasticity, and other excellent physical properties [8,9], such as conveyor belts, sponge products, gas masks, various tires, medical gloves, warm bags printing special parts, and other latex products [10,11,12]. With the rapid development of the economy and the improvement in human living standards, the demand for natural rubber is increasing [13]. At present, *Hevea brasiliensis* is almost the primary commercial source of natural rubber [14]. However, the rubber tree as a single species is at risk, with a long breeding season, limited planting area, and vulnerability to potential threats from climate change and disease [15,16,17]. Therefore, to increase the yield of natural rubber, it is necessary to develop an alternative source of natural rubber [18,19]. 

*Taraxacum kok-saghyz* Rodin (TKS), also known as Russian dandelion, is a self-incompatible perennial herbaceous plant of the family *Asteraceae* [20]. As a promising rubber-producing crop, it has the advantages of a short growth cycle, high adaptability, wide cultivation area, relatively simple genome, easy reproduction, and mature genetic transformation system [21,22]. Furthermore, high molecular weight natural rubber can be produced in the laticifers of TKS roots [23,24], and its rubber production is comparable to the yield of the *H. brasiliensis* [25]. In addition, TKS can also produce some valuable compounds, such as inulin, pentacyclic triterpene, and sterol; the change in their content has a certain effect on the synthesis of natural rubber [26]. 

Plants have the following two main metabolic pathways for isoprenoid biosynthesis: mevalonate (MVA) and methylerythritol (MEP). The MVA pathway mainly occurs in the cytoplasm, while the MEP pathway operates in the plastid [27]. These two pathways provide precursors for isoprene biosynthesis, changes in gene expression and enzyme activity in either pathway will affect the metabolic balance of cells and plant growth and development [28]. Geranylgeranyl pyrophosphate synthase (GGPS) serves as an essential enzyme in the metabolism of isoprenoids [29], catalyzing the synthesis of 20-carbon geranylgeranyl diphosphate (GGPP) from isopentenyl pyrophosphate (IPP) and its isomer dimethylallyl pyrophosphate (DMAPP), with the substrate being provided by the MVA or MEP pathways [30]. In addition, GGPP is a significant intermediate compound in the bio-production of various isoprenoids, such as natural rubber, carotene, gibberellin, tocopherol, and cholesterol [31,32]. The *GGPS* genes have been studied in certain plants so far. Heterologous expression of the sunflower genes (*HaGGPS*) on chloroplasts in tobacco and *Arabidopsis thaliana* results in rapid growth, early flowering, and an increase in gibberellin (GA) content and the number of flowers and seed pods [33]. The identification of the *GGPS* gene family in several cotton varieties and the expression analysis of *GGPSs* in plant development and abiotic stress indicated that *GGPS* participated in chlorophyll synthesis of cotton, promoted fiber development, and responded to abiotic and hormonal stress. [34,35]. The cloning and expression analysis of the *GGPS1* gene in *Salvia miltiorrhiza* showed that the *GGPS1* gene was expressed in all tested tissues, but its expression level was higher in leaves at the flowering stage [36]. The gene cloning, expression, and function analysis of GGPS synthase from *H. brasiliensis* indicated that GGPS synthase in *H. brasiliensis* rubber latex may be involved in natural rubber and β-carotene biosynthesis [32]. Twelve *GGPS* synthase gene family members were predicted in *A. thaliana*, suggesting that some key genes are provided for specific tissues, developmental stages, or metabolic pathways [37]. However, the identification and characterization of *GGPS* gene family members have not been reported in *T. kok-saghyz*. 

In this study, we comprehensively identified seven *GGPS* family members in TKS. First of all, we analyzed the physicochemical properties, secondary and tertiary structures of TkGGPS proteins, and performed bioinformatics analysis of conserved motif and domain, gene structure, chromosome localization, inter-specific phylogenetic relationship, and collinearity features. Then, we investigated the expression of *TkGGPS* genes in different tissues and under hormone treatments by analyzing transcriptomic data and qRT-PCR. Finally, we determined the subcellular localization of all TkGGPS proteins. The results obtained in this study will contribute to a deeper understanding of the role of *TkGGPSs* in the growth and development and the biosynthesis of natural rubber in *T. kok-saghyz*.

## 2. Results

### 2.1. Identification of the GGPS Gene Family in T. kok-saghyz

A total of seven *GGPS* genes were identified based on the latest genomic datas by local BLAST analysis and HMM search with the GGPS protein sequence of *Arabidopsis thaliana* as a query. We also selected *Arabidopsis thaliana*, a closely related and rubber-producing related species to further characterize the *TKGGPS* gene family using an orthology-based method. As a result, six TkGGPS members were identified, which did not include TkGGPS5 (Supplementary Tables S1 and S2), so we compared the structure and physicochemical properties of the TkGGPS5 protein with those of several other members and found that this member shared similar structure and properties with the others; therefore, we classified *TkGGPS5* as a member of the *TkGGPS* gene family.

The seven TkGGPS members were named according to the length of their amino acid sequences, and their physicochemical properties were analyzed, as shown in Table 1. The length of the TkGGPS amino acid sequences ranged from 262 aa (TkGGPS1) to 436 aa (TkGGPS7), the molecular weights ranged from 28.53 kDa (TkGGPS2) to 48.34 kDa (TkGGPS7), their predicted isoelectric points ranged from 5.14 (TkGGPS3) to 9.76 (TkGGPS5), and the instability index ranged from 34.27 (TkGGPS2) to 62.03 (TkGGPS1). All GGPS proteins were expected to be localized on chloroplasts, and most members of this family had an instability index of more than 40, indicating that they are unstable. The GRAVY ranged from −0.247 (TkGGPS5) to 0.096 (TkGGPS6), most TkGGPS members have more hydrophilic regions than hydrophobic regions (Supplementary Figure S1), while the average hydrophobicity of TkGGPS 2 and TkGGPS 6 was 0.038 and 0.096, respectively, suggesting that they may be hydrophobic proteins. In addition, the trans-membrane domain analysis of the amino acid sequence of TkGGPSs (Supplementary Figure S2) showed that all the amino acids of these seven TkGGPS members were outside the membrane, indicating that TkGGPS protein synthesis could play a direct role without transmembrane transport. The prediction and analysis of signal peptides of seven TkGGPS proteins (Supplementary Figure S3) showed that there were no signal peptides in TkGGPS proteins, and they were non-secreted proteins.

### 2.2. Secondary and Three-Dimensional Structures Analysis of the TkGGPSs

The secondary structure of the seven TkGGPS proteins was predicted and analyzed, and the results are shown in Table 2. The α-helices and random coils are the main structures; there is no β-folding structure, but the extension chain and β-turn are scattered throughout the protein. Among them, the α-helix occupies 42.12% (TkGGPS5)–63.26% (TkGGPS2), while the β-turn occupies 3.67% (TkGGPS7)–9.09% (TkGGPS5), the extended strand occupies 4.92% (TkGGPS2)–16.97% (TkGGPS5), and the random coil chain accounts for 25.37% (TkGGPS3)–38.30% (TkGGPS7).

**Figure 1:**
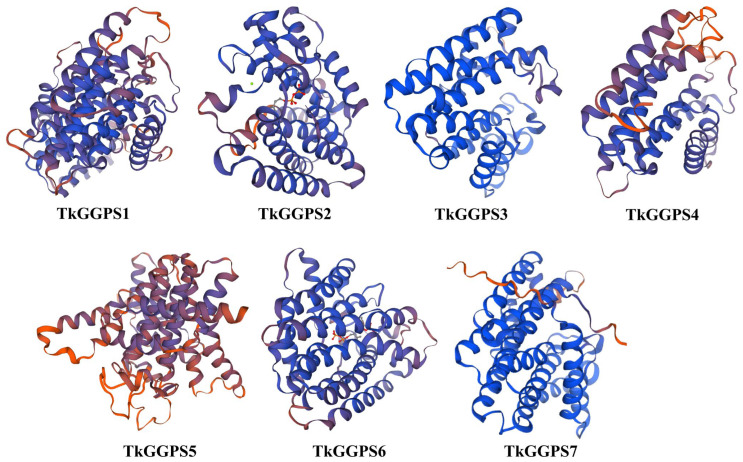
Prediction of the three-dimensional structure of TkGGPS proteins in *T. kok-saghyz*. The blue chain represents consistency with the template sequence and a higher score, while the red represents a lower score.

The three-dimensional structure of the seven TkGGPS proteins was predicted by homology modeling, and the results are shown in Figure 1. The similarity between TkGGPS proteins and the template sequence ranges from 34.05% (TkGGPS5) to 87.12% (TkGGPS7). It can be observed that the three-dimensional structures of the TkGGPS proteins are predominantly composed of α-helices and random coils, resulting from further twisting and folding processes. From a three-dimensional structure perspective, there is a significant resemblance among the different members of the TkGGPSs. Therefore, it can be inferred that these members likely share similar protein functions in this plant. Only TkGGPS5 has a different three-dimensional structure from the other members, which may be due to its differentiation during evolution.

### 2.3. Conserved Motif, Domain and Gene Structure of the TkGGPS in T. kok-saghyz

**Figure 2:**
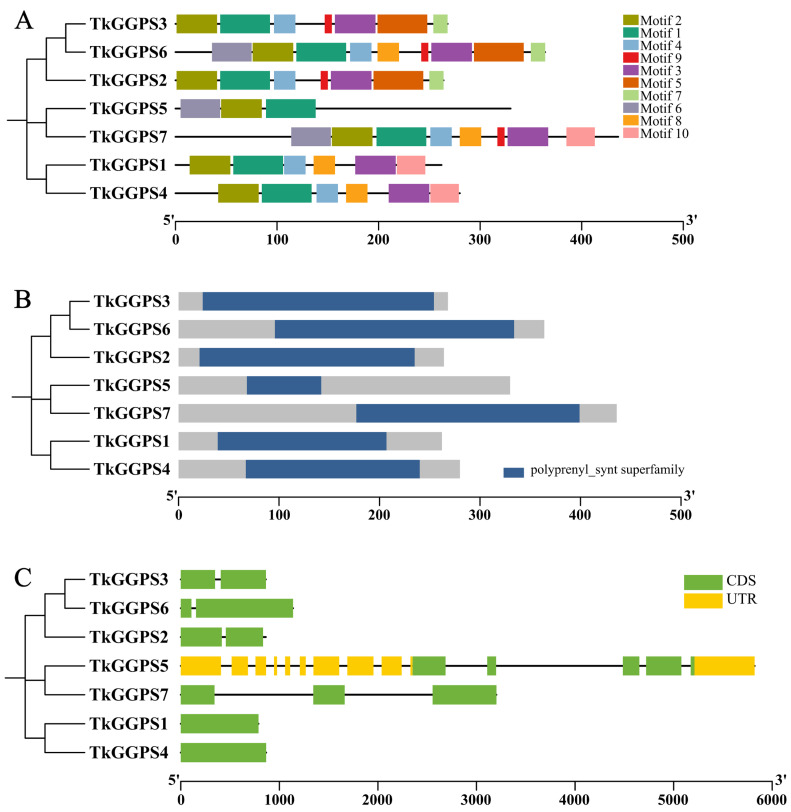
Phylogenetic relationship, motif composition, conserved domains, and gene structure of the TkGGPS family proteins. (**A**) The phylogenetic tree and the motif composition in TkGGPSs. Different colored boxes represent putative motifs, and the lengths of motifs in each protein are presented proportionally. The phylogenetic tree was constructed using the Neighbor-Joining (NJ) method. (**B**) The polyprenyl_synt superfamily domain of TkGGPSs. (**C**) The exon–intron structure of the *TkGGPS* genes. Yellow boxes indicate 5′ UTR and 3′ UTR, green boxes indicate exons, black lines indicate introns.

The MEME and Pfam websites were employed to identify the conserved motifs and domains of these TkGGPSs (Figure 2). A total of ten motifs were identified. TkGGPS2, TkGGPS3, and TkGGPS6, TkGGPS5 and TkGGPS7, TkGGPS1 and TkGGPS4, respectively, from the same clade shared common motif compositions (Figure 2A). Conserved motifs in the TkGGPSs indicated that almost all the TkGGPSs contained motifs 1 and 2. However, TkGGPS6 lacked motif 10, TkGGPS7 lacked motifs 5 and 7, while TkGGPS2 and TkGGPS3 lacked motifs 6, 8, and 10. TkGGPS1 and TkGGPS4 lacked motifs 5, 6, 7, and 9. Motif 9 was present in TkGGPS2, TkGGPS3, TkGGPS6, and TkGGPS7. TkGGPS5 contained motifs 1, 2 and 6. All TkGGPSs contained one polyprenyl_synt superfamily domain, which was typical of a GGPS protein (Figure 2B). 

To gain further insights into the structural diversity of TkGGPSs, the exon/intron organizations of TkGGPS genes were determined by Tbtools software. Most TkGGPS genes contained exons and introns, but TkGGPS1 and TkGGPS4 have no intron (Figure 2C). The multiple sequence alignment results revealed a partial or total variation in the five key residues of all seven TkGGPSs (Supplementary Figure S4). The members of the TkGGPS include five conserved amino acid regions named I, II, III, IV, and V. The first conserved region is GKXXR (X is an arbitrary amino acid), the third region is GQ, and the fourth region is KT. In addition, the second and fifth regions are two aspartate-rich regions, DDXXXXD and DDXXD, that are called the FARM (the first aspartate-rich motif) and SARM (the second aspartate-rich motif) regions, which are the binding sites of allyl substrate and IPP, respectively.

### 2.4. Cis-Acting Elements Analysis of the TkGGPS Gene Family

**Figure 3:**
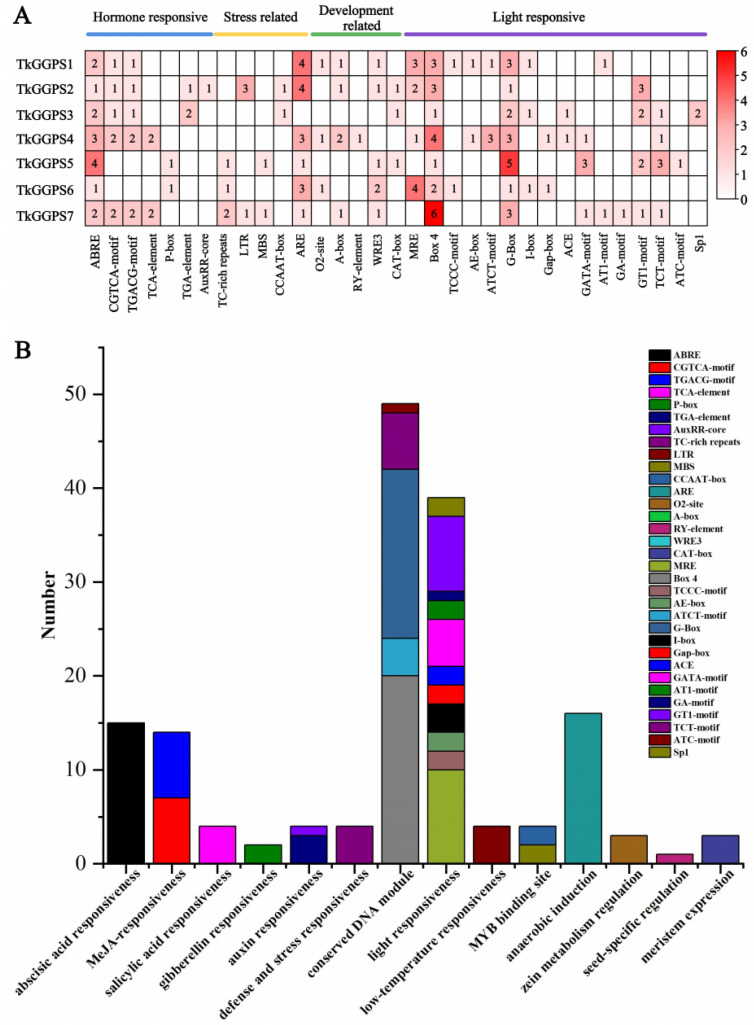
Predicted cis-acting elements of the promoters of *TkGGPSs*. (**A**) Classification and heatmap expression analysis of all cis-acting elements on promoters of *TkGGPSs*. The 2.0 kb promoter sequences of seven *TkGGPS* genes were analyzed using the PlantCARE database. The different colors represent the number of each cis-acting element. (**B**) Statistics of all cis-acting elements on promoters of *TkGGPSs*.

Cis-acting elements are widely recognized as crucial factors in gene adaptation to environmental changes and the regulation of growth and development. Therefore, to gain preliminary insights into the role of the seven *TkGGPS* genes, we analyzed the potential cis-acting elements in their promoter regions. A comprehensive examination identified a total of 33 types of cis-acting elements (Figure 3). 

These cis-acting elements were further divided into the following four functional categories: hormone response, stress response, growth and development, and light response (Figure 3A). For the hormone-responsive category, the abscisic acid-responsive element accounted for the largest proportion (ABRE7/7), followed by the MeJA-responsive elements (CGTGA-motif, 5/7, TGACG-motif, 5/7), auxin-responsive elements (TGA-element, 2/7, AuxRR-core, 1/7), salicylic acid-responsive element (TCA-element, 2/7), and gibberellin-responsive element (P-box, 2/7). For the stress-responsive category, six *TkGGPSs* contained the anaerobic induction element (ARE), three *TkGGPSs* owned defense and stress responsiveness elements (TC-rich repeats), two *TkGGPSs* possessed the low-temperature responsiveness element (LTR), two *TkGGPSs* had the binding site MYBHv1 element (CCAAT-box), and two *TkGGPSs* had the drought-inducibility element (MBS). For the developmental regulation category, WRE3 related to developmental regulation was the most abundant element (5/7), followed by A-box related to developmental regulation (4/7), CAT-box involved in meristem expression (3/7), O_2_-site involved in zein metabolism regulation (3/7), and RY-element involved in seed-specific regulation (1/7). Notably, within the light-responsive element category, the conserved DNA module subcategory mainly exhibited motifs corresponding to 16 types of cis-acting elements, including MRE, Box 4, TCCC-motif, AE-box, ATCT-motif, G-Box, I-box, Gap-box, ACE, GATA-motif, AT1-motif, GA-motif, GT1-motif, TCT-motif, ATC-motif, and Sp1 (Figure 3B). Interestingly, all seven *TkGGPSs* contained a large number of light-responsive elements (Figure 3A), suggesting a close association between the *TkGGPSs* and photosynthesis in TKS.

### 2.5. Phylogenetic Analysis of TkGGPS Proteins

**Figure 4:**
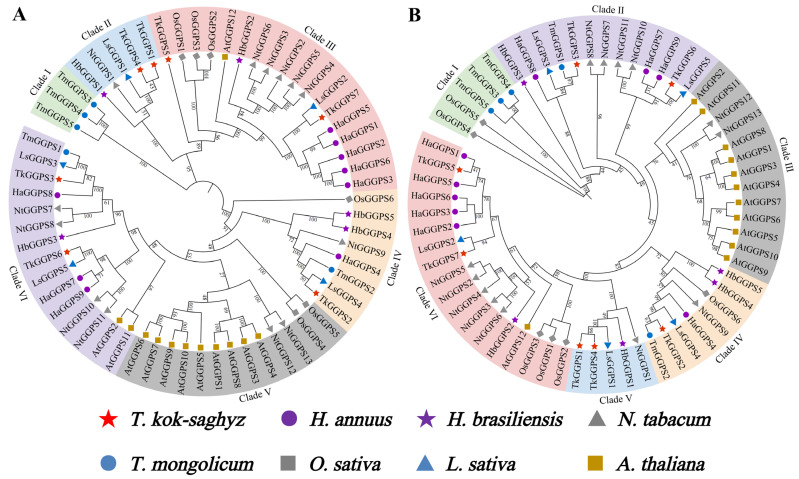
Phylogenetic analysis of TkGGPS proteins in *T. kok-saghyz*. Phylogenetic analyses were conducted on GGPS proteins from *T. kok-saghyz* (TKS, red star), *H. brasiliensis* (Hb, purple star), *L. sativa* (Ls, blue triangle), *T. mongolicum* (TM, blue circle), *N. tabacum* (Nt, dimgray triangle), *O. sativa* (Os, dimgray square), *A. thaliana* (At, gold square) and *H. annuus* (Ha, purple circle). ClustalW was used for multiple sequence alignment. Phylogenetic trees were constructed using the Neighbor-Joining (NJ) method with 1000 bootstrap repeats (**A**) and the Maximum likelihood (ML) method with 5000 bootstrap repeats (**B**). Different colors represent different clades of GGPS. The number on the branch represents the Bootstrap method (BM) value.

To investigate the phylogenetic relationship among GGPS proteins upon representative monocotyledonous and dicotyledonous plants, we constructed phylogenetic trees with 7 TkGGPS, 5 HbGGPS, 5 LsGGPS, 5 TmGGPS, 13 NtGGPS, 6 OsGGPS, 12 AtGGPS and 9 HaGGPS proteins from eight species, namely *T. kok-saghyz*, *H. brasiliensis*, *Lactuca sativa*, *Taraxacum mongolicum*, *Nicotiana tabacum*, *Oryza sativa*, *A. thaliana*, and *Helianthus annuus*. The protein names used for AtGGPS are those known from TAIR, and the remaining GGPS names for each species are based on the length of the amino acid sequence (Supplementary Table S3). The GGPS of all the above eight species were classified into six clades using the Neighbor-Joining (NJ) and Maximum-Likelihood methods (ML), respectively (Figure 4). In the phylogenetic tree constructed by the NJ method, TkGGPS1 and TkGGPS4 belonged to clade II, TkGGPS5 and TkGGPS7 belonged to clade III, and two proteins (TkGGPS3 and TkGGPS6) belonged to clade VI (Figure 4A). Similarly, these six TkGGPS members are also in three clades (II, V, and VI) in the phylogenetic tree constructed with the ML method (Figure 4B). However, TkGGPS2 is in a different cluster from the other TKS members in both phylogenetic trees, so it was hypothesized that TkGGPS2 may play a different role. Interestingly, most of the *A. thaliana* members are in the same cluster, which was presumably more evolutionarily conserved and with similar functions.

### 2.6. Location and Collinearity Analysis of TkGGPS Genes on chromosomes

**Figure 5:**
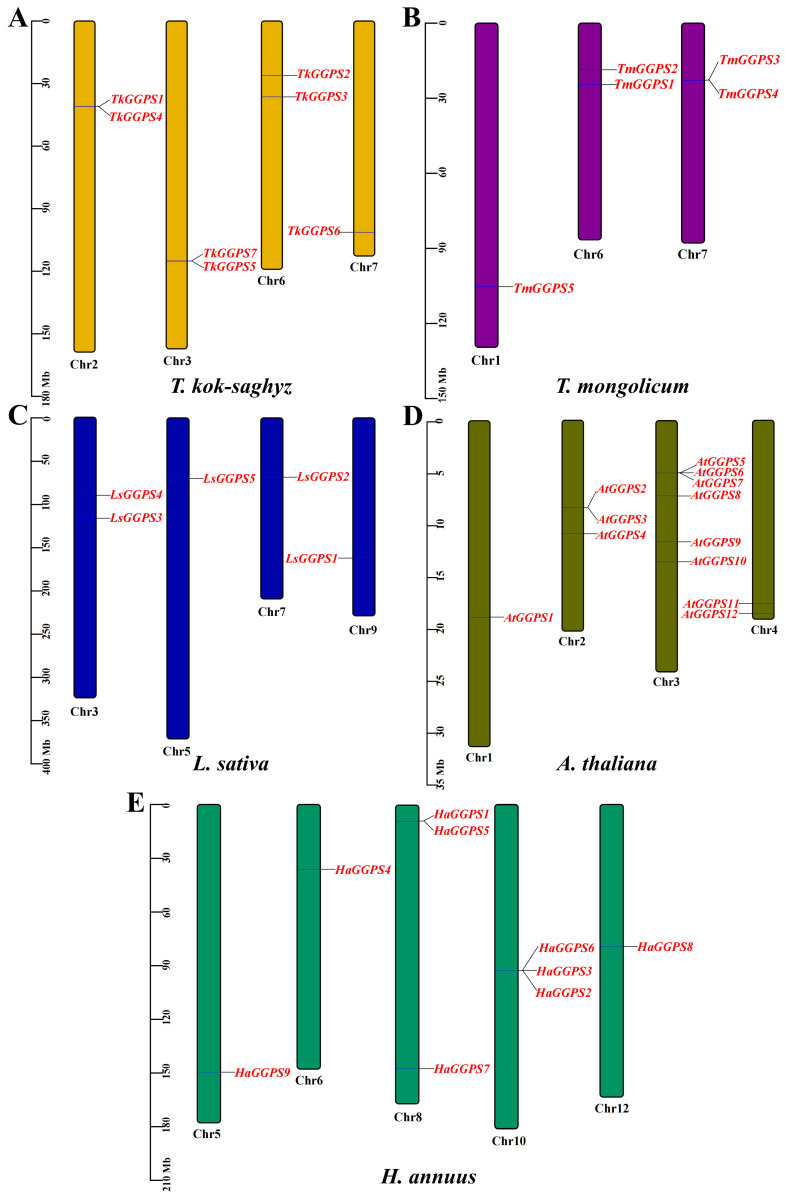
Chromosome Distribution of *GGPS* genes in the genomes of five species. (**A**) *T. kok-saghyz*, (**B**) *T. mongolicum*, (**C**) *L. sativa*, (**D**) *A. thaliana*, and (**E**) *H. annuus*. The scale on the left represents the chromosome size, the length of chromosomes is measured in Mb. The chromosome number is indicated below each chromosome. *GGPS* gene numbers are shown on the two sides of each chromosome, and they are marked in red.

To observe whether the members of the *TkGGPS* gene family are distributed in clusters on chromosomes, the *GGPS* localizations of *T. kok-saghyz* and four similar species were investigated, and the results are shown in Figure 5. A total of eight chromosomes in TKS, of which seven members of *TkGGPS* were distributed on chromosomes 2, 3, 6, and 7. *TkGGPS1* and *TkGGPS4* were located on chromosome 2. *TkGGPS5* and *TkGGPS7* were located on chromosome 3, and *TkGGPS2* and *TkGGPS3* were located on chromosome 6. Only *TkGGPS6* was located on chromosome 7 (Figure 5A). In addition, there were a total of eight chromosomes in *T. mongolicum*, and the *TmGGPS* members were distributed on chromosomes 1, 6, and 7 (Figure 5B). There were nine chromosomes in *L. sativa*, among which the *LsGGPS* family members were distributed on chromosomes 3, 5, 7, and 9, respectively (Figure 5C). There were five chromosomes in *A. thaliana*, and members of the *AtGGPS* family were distributed on chromosomes 1, 2, 3, and 4 (Figure 5D). In H. *annuus*, there were 17 chromosomes, and the *HaGGPS* members were distributed on chromosomes 5, 6, 8, 10, and 12 (Figure 5E).

**Figure 6:**
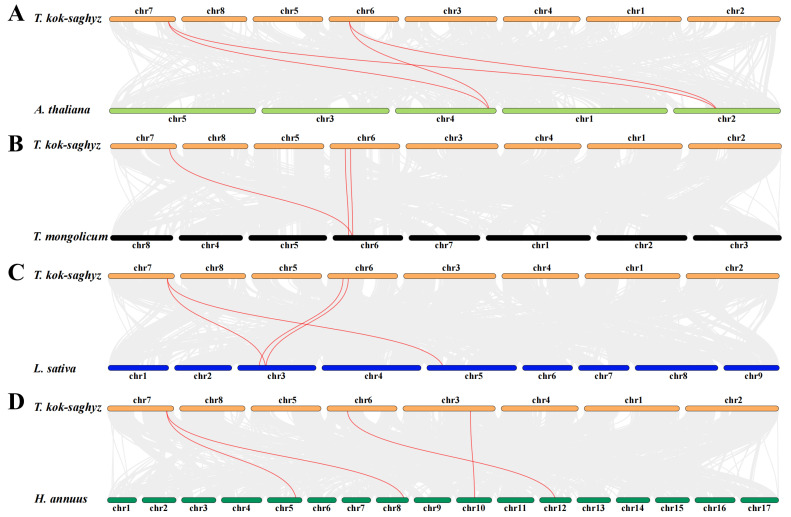
Collinearity analysis of *TkGGPS* genes. Genome collinearity features between (**A**) *T. kok-saghyz* and *A. thaliana*, (**B**) *T. kok-saghyz* and *T. mongolicum*, (**C**) *T. kok-saghyz* and *L. sativa*, (**D**) *T. kok-saghyz* and *H. annuus*. All the collinear *GGPS* genes are shown in red lines. The grey lines indicate collinear blocks.

To assess the collinearity relationships of the *TkGGPS* gene family within the various common model crops, we analyzed the molecular phylogeny of the GGPS gene family using the One Step MCScanX—Super Fast toolkit in TBtools software, as shown in Figure 6. The results showed that *T. kok-saghyz* exhibited three collinear gene pairs with *T. mongolicum* and four collinear gene pairs with *A. thaliana*, *L. sativa*, and *H. annuus*, respectively. Notably, *TkGGPS3* and *TkGGPS6* have homologous gene pairs with all four species (Supplementary Table S4), and *TkGGPS7* and *HaGGPS6* are homologous genes. In addition, *TkGGPS2* is homologous with *LsGGPS4* and *TmGGPS2*, respectively, suggesting that they may play similar roles in different species.

### 2.7. Transcriptomics and qRT-PCR Analysis of TkGGPS Genes

**Figure 7:**
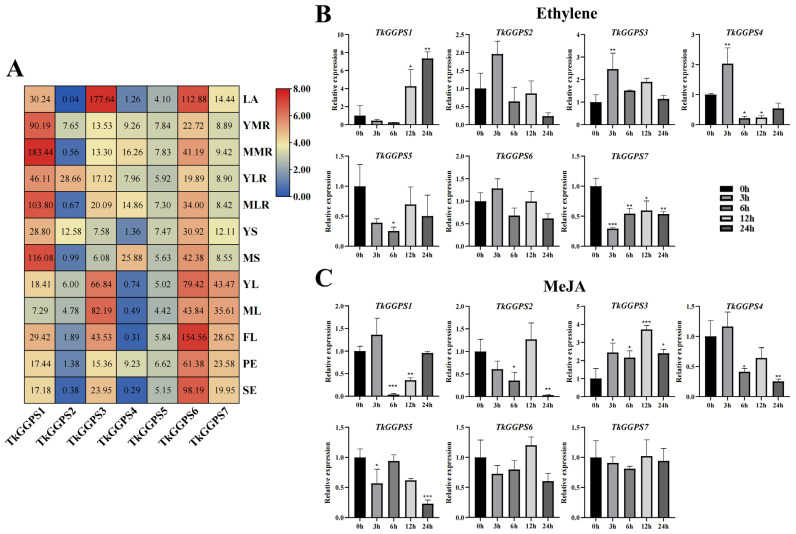
Gene expression of 7 *TkGGPS* genes in TKS. (**A**) Heat map expression analysis of *TkGGPS* genes in TKS different tissues based on transcriptome data. LA, latex; YMR, young main root; MMR, mature main root; YLR, young lateral root; MLR, mature lateral root; YS, young stem; MS, mature stem; YL, young leaf; ML, mature leaf; FL, flower; PE, peduncle; SE, seed. Red indicates high expression levels, and blue indicates low expression levels. The numbers on the box represent the fragments per kilobase million (FPKM) value. The expression values were normalized by FPKM to create the heat map, using the average FPKM of three biological replicates. The expression level of 7 *TkGGPS* genes in TKS roots treated with ethylene (**B**) and MeJA (**C**) at 0 h (control), 3 h, 6 h, 12 h, and 24 h, respectively. Data were normalized to β-actin. Vertical bars for qRT-PCR indicate the standard deviation, while an asterisk indicates the summary *p*-value of the independent samples t-test of the corresponding gene compared to the control (* *p* < 0.05, ** *p* < 0.01, *** *p* < 0.001).

Since natural rubber is synthesized in latex from TKS roots, we performed transcriptome analysis to explore the expression patterns of the seven *TkGGPS* gene members in 12 different TKS tissues including latex (Figure 7A). We found that all seven *TkGGPS* members were expressed in latex, with *TkGGPS1* having a relatively high expression, and *TkGGPS3* and *TkGGPS6* both having very high expressions, with a more than one-hundred-fold expression, whereas *TkGGPS2*, *TkGGPS4*, and *TkGGPS7* had a low expression. We hypothesized that *TkGGPS1*, *TkGGPS3*, and *TkGGPS6* may play an important role in NR synthesis. In addition, this result indicated that the *TkGGPS2* expression was higher in young lateral roots compared to other tissues, while the *TkGGPS1* gene was highly expressed in main roots, lateral roots, and stems. *TkGGPS6* expression was generally high, and *TkGGPS5* expression was more stable in all the tissues tested. Furthermore, *TkGGPS3* and *TkGGPS7* expression was high in the young leaves, mature leaves, flowers, peduncles, and seeds, whereas the expression pattern in roots showed a more stable trend. It indicated that they play a part in the plant growth and developmental processes.

To understand the function of the *TkGGPS* gene members in plant development, we used different hormones (ethylene and MeJA) to treat a 6-month-old wild-type TKS and analyzed it by qRT-PCR to probe the expression levels of seven *TkGGPSs* in the roots and leaves. The expression of the seven *TkGGPS* genes was different in response to these two hormones, as shown in Figure 7. The expression of *TkGGPS3* was up-regulated while two genes (*TkGGPS5* and *TkGGPS7*) were down-regulated (compared to the control) by ethylene treatment in TKS roots (Figure 7B). After ethylene treatment for 3 h, the expressions of *TkGGPS2*, *TkGGPS4*, and *TkGGPS6* were up-regulated and then down-regulated. At 12 h and 24 h, and the expression level of *TkGGPS1* was up-regulated, and the accumulation was up to seven times at 24 h, thus we speculated that ethylene could regulate gene expression. However, the expression levels of two genes (*TkGGPS2* and *TkGGPS3*) had little response to ethylene treatment in the TKS leaves; *TkGGPS6* was up-regulated, while *TkGGPS4* and *TkGGPS5* were down-regulated (Supplementary Figure S5A). After treatment with MeJA, the expression levels of *TkGGPS1* and *TkGGPS4* in TKS roots were up-regulated at 3 h, whereas those of *TkGGPS2* and *TkGGPS6* were up-regulated at 12 h. The expression levels of the *TkGGPS5* and *TkGGPS7* genes were largely unaffected by MeJA (Figure 7C). Similarly, after MeJA treatment of leaves, the response of *TkGGPS5* and *TkGGPS7* to MeJA treatment remained small, and the expression of the *TkGGPS3* gene was still up-regulated, demonstrating that *TkGGPS3* plays an important role in the regulation of the growth and development of TKS as well as the metabolic pathways of ethylene and MeJA (Supplementary Figure S5B).

### 2.8. Subcellular Localization Analysis of TkGGPS Proteins

**Figure 8:**
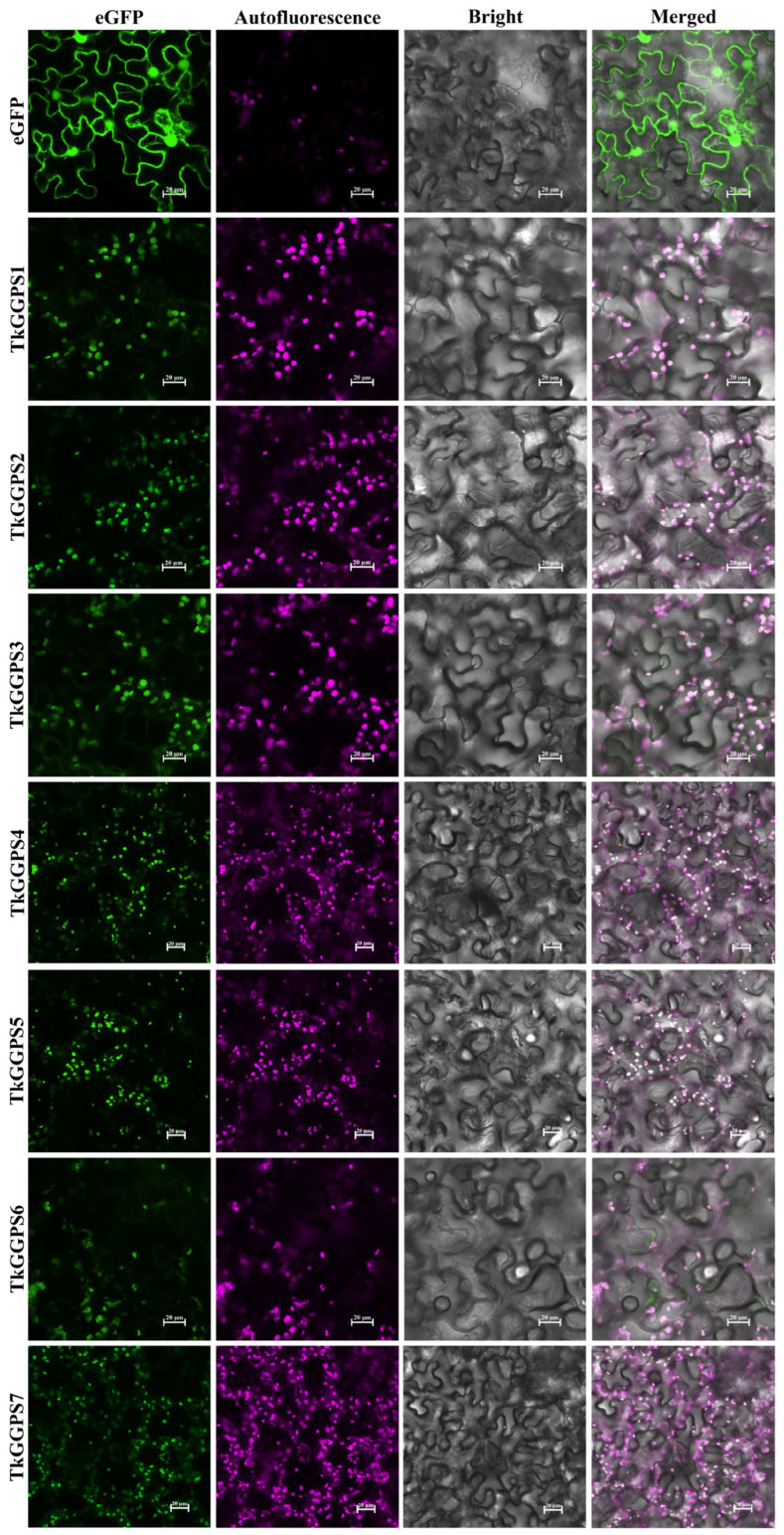
Subcellular localization of TkGGPS proteins in *Nicotiana tabacum* leaves. pCAMBIA1300-35S–eGFP and pCAMBIA1300-35–TkGGPS–eGFP fusion proteins were transiently expressed in *N. tabacum* leaves. The fields included green fluorescence filed (488 nm), chloroplast autofluorescence field (640 nm), bright field, and merged filed. Empty vector control showing the expression of 35S–eGFP in epidermal cells of *N. tabacum*, and co-localization of 35S–eGFP with TkGGPS proteins observed by chloroplast autofluorescence. Bars = 20 µm.

To determine the subcellular localization of the seven TkGGPS proteins, green fluorescent protein (eGFP)-expressing vector-fused TkGGPS proteins were constructed, and this plasmid was transiently expressed in tobacco leaf epidermal cells. As shown in Figure 8, the signals of 35S–TkGGPS–eGFP are found in the chloroplast. Additionally, subcellular localization prediction analysis revealed that most of the TkGGPS proteins are predominantly localized in the chloroplast. These localization patterns are consistent with the expected properties of the theoretical prediction. The above results suggest that TkGGPS members are not only present in the latex of the TKS roots involved in NR synthesis but also in the chloroplasts involved in plant photosynthesis.

## 3. Discussion

Geranylgeranyl pyrophosphate synthase (GGPS) is a key enzyme in the synthesis of GGPP and is widely found in plants, animals, bacteria, and fungi. It catalyzes the condensation reaction of farnesyl pyrophosphate (FPP) and isopentenyl pyrophosphate (IPP) to produce GGPP, which is an important precursor for the synthesis of natural rubber [38]. NR is synthesized in the roots of TKS and stored in rubber particles, which has important application value in human life [39]. At present, the *GGPS* gene has only been reported in a few plants, such as sweet potato [40], *Taxus media* [41], *A. thaliana* [42], *N. tabacum* [43], and so on. However, the identification and analysis of the *GGPS* gene family is rarely studied in *T. kok-saghyz*. Through previous transcriptomics and proteomic studies, GGPS was found to be important for NR biosynthesis in small rubber particles in TKS roots [44]. Here, we identified and verified a total of seven members of the *TkGGPS* gene family based on the newly published genome by Lin et al. [45]. In this publication, Lin et al. researchers found that the contents of GPP and GGPP in TKS latex were high. Therefore, it is necessary to explore the molecular characteristics and regulation mechanism of the *TkGGPS* gene family. In this study, the physicochemical property, conserved motif, domain, gene structure, cis-acting element, phylogenetic relationship, chromosome localization, and collinearity of the *TkGGPS* gene family members were analyzed, which is of great significance for further understanding the molecular characteristics of the *GGPS* genes in TKS.

The amino acid sequence of a protein determines how its higher structure is formed as well as its ultimate function [46], so it is imperative to analyze the physicochemical properties of a protein. The number of amino acids, the molecular weight, and the isoelectric point of the TkGGPS members were described in this study (Table 1). Most members of TkGGPS are hydrophilic, have no transmembrane domains, no signal peptides, and are unstable, non-secreted proteins (Supplementary Figures S1–S3). α-helix and random curling are the main components of the secondary and tertiary structure of TkGGPS proteins (Table 2, Figure 1). Conserved motifs and domains are usually associated with the function of proteins [47]. By analyzing the motif and domain structures, we found that although several motifs are missing in TkGGPS, some motifs are present in all members of TkGGPS, such as motif 1 and motif 2 (Figure 2A). It was noteworthy that the polyprenyl_synt superfamily domain was present in all the TkGGPS members (Figure 2B), suggesting that they are highly conserved in TkGGPS and may play similar roles in plant growth and development. Introns are genomic sequences removed from the corresponding RNA transcripts of genes and play an important role in species’ genetic and evolutionary processes. In earlier reports, introns of some genes were lost over time during gene replication [48]. Among the seven *TkGGPS* members in this study, introns were missing in some genes, such as *TkGGPS1* and *TkGGPS4*, which may be related to their evolution (Figure 2C). Multiple sequence alignment results (Supplementary Figure S4) showed that there are two aspartate-rich motifs in the TkGGPS members, FARM and SARM. The FARM motif is sequenced as DDxxD or DDxxxxD, while the SARM motif is always DDxxD; both motifs bind three Mg^2+^ ions, which is conducive to the binding of IPP and DMAPP to GGPS substrates and determines the catalytic activity of GGPS [49,50].

Phylogenetic relationships are used to represent the relationship between species or amino acid sequences, and accurate phylogenetic trees can increase our understanding of evolutionary relationships [51]. In the present study, all GGPSs were grouped into a broad category, the phylogenetic tree divides the GGPSs of eight species into six clades (Figure 4). It was concluded that the TkGGPS members in the same clade have similar gene structure, conserved domain, and motif composition (Figure 2). Collinear relationships between species are common in nature, and homologous genes perform the same function in the different species [52]. Here, *TkGGPS* genes showed good collinearity with *T. mongolicum*, *L. sativa*, and *H. annuus*, which belong to dicotyledonous plants in the *Asteraceae* family. In particular, the *TkGGPS3* and *TkGGPS6* genes on chromosomes 6 and 7 of *T. kok-saghyz* shared homologous genes with four other species (Figure 5 and Figure 6), which were predicted to have potentially evolved from a common ancestor, with a high degree of conservation and a slower evolutionary rate.

Transcriptomics provides comprehensive access to transcript information for species-specific tissues or organs [53]. Here, almost all the gene members of *TkGGPS* were expressed in TKS latex (Figure 7A), where NR is synthesized. In the previous study, a total of 102 rubber biosynthesis-related genes were identified by genome assembly, including 19 genes for initiator synthesis [54]. It was shown that the NR initiators—the GPS, FPS, and GGPS enzymes—can catalyze IPP and DAMPP to generate isopentenyl diphosphate intermediates such as GPP, FPP, and GGPP, thereby initiating rubber biosynthesis in vitro [55]. In this study, the expression of *TkGGPS1* was higher in the TKS roots and latex than in the other tissues examined, such as the leaves and flowers, while the expression of the *TkGGPS3* and *TkGGPS6* genes was significantly higher in latex than in the other gene members, which were in agreement with the conclusion drawn by Lin et al. [45]. We speculated that these three genes could be candidate genes and may play a key role in NR synthesis. In addition, all the *TkGGPS* gene members were differentially expressed in various tissues in *Taraxacum kok-saghyz*, which may be related to their role and specific function in each tissue.

Ethylene and MeJA are widely involved in regulating plant growth and development and secondary metabolite synthesis [56]. Cis-acting elements are involved in the regulation of gene expression and are necessary for plant growth, adaptation, and response to the environment [57]. In this study, we predicted the cis-acting elements of the *TkGGPS* gene members, including hormone-responsive elements, stress-responsive elements, growth- and development-responsive elements, and light-responsive elements (Figure 3). It has been reported that exogenous MeJA can activate JA biosynthesis and signal transduction and regulate the downstream MEP, MVA, and natural rubber biosynthesis pathways by altering gene expression [58]. Exogenous ethylene can promote seed germination and rooting [59], and exogenous methyl jasmonate can induce *CgGGPS* expression in *Corylus avellana* L. Gasaway [60]. Therefore, we treated different tissues of TKS with ethylene and MeJA and performed qRT-PCR to detect the expression level of the *TkGGPS* genes (Figure 7); we found that these two hormones induced the expression of *GGPS* genes in TKS roots, and the expression levels of *TkGGPS3* were always up-regulated after treatment. Moreover, these two hormones also affect the expression of *GGPS* genes in the TKS leaves (Supplementary Figure S5). Therefore, the expression of the *TkGGPS* gene members was regulated by the ethylene and MeJA pathways, and these members had different expression levels after hormone induction.

The subcellular localization of GGPS is significantly differentiated in plants to accommodate its different physiological functions [61]. In plants, the *GGPS* gene was first successfully cloned in *Capsicum annuum*, and the in situ immunolocalization experiment proved that the CaGGPS protein was only located in plastids. Furthermore, it was heavily induced in the process of chloroplast to chromoplast transformation in mature fruit, and the corresponding enzyme activity was also enhanced [62]. In *A. thaliana*, a total of twelve members of the AtGGPS family were identified in earlier studies, among which AtGGPS1 was located in the mitochondria, AtGGPS3 and AtGGPS4 in the endoplasmic reticulum, and AtGGPS2, AtGGPS6, AtGGPS7, and AtGGPS11 in the chloroplasts or plastids [63]. In rice, OsGGPS1 is mainly located in the plastoglobules and stroma of chloroplasts [64]. In *Liriodendron tulipifera*, the LtGGPS2 protein is localized to the chloroplasts, suggesting that this gene may be involved in carotenoid and chlorophyll synthesis [65]. In *H. brasiliensis*, HbGGPSs were detected in all the tissues examined, including the latex, leaves, flowers, and pedicels, which may participate in the synthesis of a variety of compounds [32]. In this study, we predicted similar results for all seven TkGGPS protein members. Most genes such as *TkGGPS1*, *TkGGPS3*, *TkGGPS6*, and *TkGGPS7* were highly expressed in stems, leaves, and flowers (Figure 7A), and the expression of certain genes was up-regulated after the treatment of TKS leaves with ethylene and MeJA (Supplementary Figure S5), suggesting that the *TkGGPS* genes were also more active in the chloroplasts. Plants generally do not contain chloroplasts in mature latex [66]; therefore, in terms of subcellular localization, it was not surprising to find that almost all the TkGGPS members were located on chloroplasts (Figure 8). Based on all the above analysis, we surmised that the *TkGGPS* gene may be involved in the biosynthesis of NR in latex and important isoprenoids such as carotenoids, chlorophylls and gibberellins in chloroplasts, which will provide us with some assistance in the subsequent validation of its function.

## 4. Materials and Methods

### 4.1. Plant Materials and Growth Conditions

TKS was collected from the river area of Shihezi City, Xinjiang, China, and successfully transplanted and cultivated in our laboratory, Shihezi University. Then, tissue culture seedlings of TKS were cultivated in the phytotron (temperature 23 °C, air humidity 68%, light 16 h) consisting of a 3:1:1 mixture of nutrient soil, vermiculite, and perlite for 5–6 months until flowering.

### 4.2. Identification and Characterization Analysis of the TkGGPS Gene Family

To identify GGPSs from the TKS genome, the 15 GGPS protein sequences in *A. thaliana* were downloaded from NCBI (https://www.ncbi.nlm.nih.gov/, accessed on 5 October 2023) and invoked as queries to perform local BLAST and HMM algorithm-based searches [67,68] against *T. kok-saghyz* and *T. mongolicum* genome databases from the National Genomics Data Center (NGDC) Genome Warehouse (GWH, https://ngdc.cncb.ac.cn/gwh/, accessed on 5 October 2023, (BioProject accession: PRJCA005187) [69]. In addition, other species’ genome databases in this study were downloaded from NCBI (https://www.ncbi.nlm.nih.gov/, accessed on 6 October 2023), such as *A. thaliana* (assembly TAIR10.1) (Supplementary Tables S7 and S8) [31], *N. tabacum* (assembly Ntab-TN90), *L. sativa* (assembly Lsat_Salinas_v11), *H. annuus* (assembly HanXRQr2.0-SUNRISE), *H. brasiliensis* (assembly ASM165405v1), and *O. sativa* (assembly GCF_001433935.1). We also selected the above eight species to further characterize the *TKGGPS* gene family by orthology-based method (https://github.com/davidemms/OrthoFinder, accessed on 6 October 2023) [70,71]. The functional domain prediction (Accession cl46106) was performed using the conserved domain tool of NCBI CDD (https://www.ncbi.nlm.nih.gov/Structure/bwrpsb/bwrpsb.cgi, accessed on 7 October 2023) [72]. To validate the accuracy of the conserved domains, these potential sequences (Pfam number (PF00348)) were further searched against the Pfam database (http://pfam-legacy.xfam.org/, accessed on 7 October 2023) [73].

In addition, the basic physicochemical properties, such as amino acid sequence length, protein molecular weight (Mw), theoretical isoelectric point (pI), and hydrophilicity of TkGGPSs, used the ExPASy website (https://web.expasy.org/compute_pi/, accessed on 10 October 2023) [74] for analysis. Prediction of the signal peptides and transmembrane domains of TkGGPS proteins used the Signal 6.0 (https://services.healthtech.dtu.dk/services/SignalP-6.0/, accessed on 13 October 2023) [75] and TMHMM 2.0 websites (https://services.healthtech.dtu.dk/services/TMHMM-2.0/, accessed on 13 October 2023) [76]. The subcellular localization of TkGGPS proteins was analyzed using the Cell-PLoc 2.0 tool (http://www.csbio.sjtu.edu.cn/bioinf/Cell-PLoc-2/, accessed on 14 October 2023) [77]. The secondary and three-dimensional structures were predicted by the online websites SOPMA (https://npsa-prabi.ibcp.fr/cgi-bin/npsa_automat.pl?page=npsa_sopma.html, accessed on 14 October 2023) [78] and SWISS-MODEL (https://swissmodel.expasy.org/interactive, accessed on 17 October 2023) [79].

### 4.3. Phylogeny, Gene Structure and Conserved Motif Analysis of TkGGPSs

First, the multiple sequence alignment of full-length GGPS amino acid sequences was performed using the ClustalW program in MEGA 11, and they were further analyzed in GeneDoc v2.7.000 software [80]. Then, the phylogenetic tree was constructed using the Neighbor-Joining (NJ) method with 1000 bootstrap replications and the Maximum likelihood (ML) method with 5000 bootstrap repeats in MEGA 11 [81]. The phylogenetic tree of GGPS proteins was beautified using EvolView v. 2 (http://evolgenius.info/evolview/#/login, accessed on 20 October 2023) [82]. Exon/intron sites and length information were extracted from the respective genome annotation GFF files of the *T. kok-saghyz* from the NGDC Genome Warehouse (BioProject accession: PRJCA005187), and the structure drawings were scaled and displayed using the Gene Structure View (Advanced) in TBtools v1.099 (Toolbox for Biologists) [83]. The conserved protein motif of the TkGGPS family was identified using MEME Suite v.5.5.5 (https://meme-suite.org/meme/tools/meme, accessed on 23 October 2023) [84], with a maximum number of 10 motifs.

### 4.4. Chromosome Distribution and Collinearity Analysis of TkGGPS Genes

Interspecific collinearity analysis and chromosomal localization were performed with TBtools. The chromosomal localization was operated by Gene Location Visualize from GTF/GFF, and collinearity analysis was conducted using the One Step MCScanX—Super Fast toolkit [85].

### 4.5. Identification of Cis-Regulatory Elements in the Promoters of TKGGPS Genes

Cis-acting elements were obtained from the 2000 bp upstream regions of the *TkGGPS* genes. The predictions were made using the PlantCARE website (http://bioinformatics.psb.ugent.be/webtools/plantcare/html, accessed on 28 October 2023) [86]. The detected elements were divided into different response types based on their annotated functions.

### 4.6. Quantitative Real-Time PCR (qRT-PCR) Analysis of TkGGPS Genes

To investigate the expression of *TkGGPS* genes in different tissues, the 6-month-old TKS seedlings with similar size and growth were selected for hormonal treatment. The seedling was hydroponic in Hogren culture solution with 100 µmol/L ethylene and 1 mmol/L MeJA hormones. Samples were collected at different treatment periods of 0 h, 3 h, 6 h, 12 h, and 24 h and packed in pre-cooled 10mL tubes, then quickly frozen in liquid nitrogen and stored in a refrigerator at −80 °C. We performed Quantitative Real-time PCR (qRT-PCR) analysis using the SYBR Green qPCR Master Mix on the Roche LightCycler 480 instrument. The reaction volume was 20 μL. The RNA was extracted according to the difficult-to-extract Plant Total RNA Kit (purchased from Magen Biotech, Guangzhou, China), and the Reverse-Transcribed cDNA Kit was used HiScript ⅡQ RT SuperMix for qPCR (+gDNA wiper) (derived from Vazyme Biotech Co., Ltd., Nanjing, China). All qRT-PCR primer sequences were designed with Primer 5.0 software (PREMIER Biosoft, San Francisco, CA, USA) and are shown in Supplementary Table S5.

To ensure the accurate normalization of the gene expression data, we used a stable and reliable reference gene, the *Tkactin* gene (GenBank accession: DY824357), as an internal control [87,88,89]. In addition, gene expression levels were quantified as relative fold changes, with the 0 h time point serving as the control group and assigned a value of one. Each reaction was conducted with three biological replicates. The obtained qRT-PCR data were analyzed using the 2^−ΔΔCT^ method, a widely accepted approach for relative quantification of gene expression [90]. The bar graph was drawn by GraphPad Prism (version 9.0, GraphPad Software, San Diego, CA, USA).

### 4.7. Transcriptomic Data Analysis of T. kok-saghyz

The transcriptomic raw data of *Taraxacum kok-saghyz* in different tissues were obtained from the Genome Sequence Archive (GSA) database (https://ngdc.cncb.ac.cn/gsa/, accessed on 15 December 2023, Accession Number: PRJCA000437). The raw data were converted into files in fastq format for quality assessment and filtered low-quality sequences [91]. Then, the obtained reads were compared to the reference genomes, and gene expression analysis was calculated according to the FPKM value [92,93]. The TBtools software was used to generate the heatmap of the *TkGGPS* genes.

### 4.8. Subcellular Localization Assay of TkGGPS Proteins

The coding sequences (CDS) of the TkGGPSs was amplified using primers that removed terminators but contained arms homologous to the expression vector, and the target fragment was recovered. Then, the sequences were cloned into the KpnI and SpeI digested pCAMBIA1300–eGFP vector by homologous recombination. Seven recombinant vectors included 35S–TkGGPS1–eGFP, 35S–TkGGPS2–eGFP, 35S–TkGGPS3–eGFP, 35S–Tk–GGPS4–eGFP, 35S–TkGGPS5–eGFP, 35S–TkGGPS6-eGFP, and 35S–TkGGPS7–eGFP, along with the empty vector 35S–1300–eGFP. The gene cloning primer sequence is shown in Supplementary Table S6.

The plasmids were introduced into *Agrobacterium tumefaciens* (strain *GV3101*) and injected into 4-week-old *N. benthamiana* leaves. Photographic observations of the tobacco leaf epidermal cells were carried out in dark culture for 48 h after transfection on a Nikon Eclipse Ti2 ultra-high-resolution confocal fluorescence microscope (Nikon, Japan). For GFP fluorescence and chloroplast autofluorescence analysis, 488/640 nm excitation laser lines were used.

## 5. Conclusions

TKS is a promising substitute crop for natural rubber, which is widely used in production and life. In this study, seven members of the *TkGGPS* gene family were identified by bioinformatics methods, and their evolutionary mechanism and functional characteristics were studied. All TkGGPS members are not only presented in latex but also located in chloroplasts. qRT-PCR analysis showed that the *TkGGPS* gene family members are also induced by ethylene and MeJA. Moreover, the transcriptome data analysis also indicated that *TkGGPS1*, *TkGGPS3*, and *TkGGPS6* would be the key candidate genes for NR synthesis, which will lay the foundation for our future research. These results provide valuable perceptions into the involvement of *TkGGPS* in natural rubber synthesis and breeding in TKS, as well as the utilization of TKS resources.

## Figures and Tables

**Table 1:** Basic information of *GGPS* genes identified in *T. kok-saghyz*.

Gene Name	Gene ID	Amino Acid	Molecular Weight (kDa)	Theoretical pI	Instability Index	GRAVY	Predicted Location
*TkGGPS1*	*GWHGBCHF009972*	262	28.83	5.53	62.03	−0.107	Chloroplast
*TkGGPS2*	*GWHGBCHF032643*	264	28.53	6.01	34.27	0.038	Chloroplast
*TkGGPS3*	*GWHGBCHF033019*	268	29.03	5.14	38.45	−0.031	Chloroplast
*TkGGPS4*	*GWHGBCHF009975*	280	31.46	6.22	59.29	−0.159	Chloroplast
*TkGGPS5*	*GWHGBCHF019242*	330	31.46	9.76	47.17	−0.247	Chloroplast
*TkGGPS6*	*GWHGBCHF039242*	364	38.82	5.16	40.19	0.096	Chloroplast
*TkGGPS7*	*GWHGBCHF019241*	436	48.34	7.58	45.50	−0.221	Chloroplast

**Table 2:** Secondary structure predictions of TkGGPSs.

Gene Name	α-Helix (%)	β-Turn (%)	Extended Strand (%)	Random Coil (%)	Structures
*TkGGPS1*	61.70	4.58	5.73	28.63	* 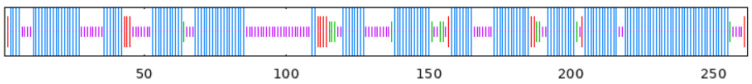 *
*TkGGPS2*	63.26	6.44	4.92	25.38	* 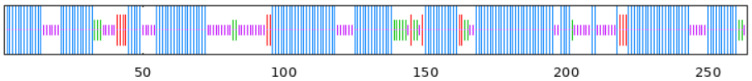 *
*TkGGPS3*	61.57	7.09	5.97	25.37	* 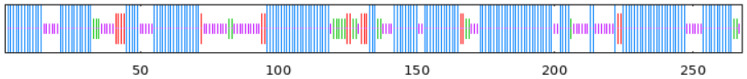 *
*TkGGPS4*	49.64	7.14	6.79	36.43	* 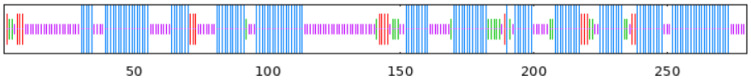 *
*TkGGPS5*	42.12	9.09	16.97	31.82	* 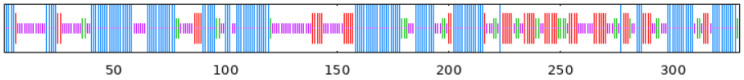 *
*TkGGPS6*	59.07	5.49	7.42	28.02	* 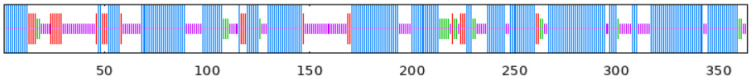 *
*TkGGPS7*	45.64	3.67	12.39	38.30	* 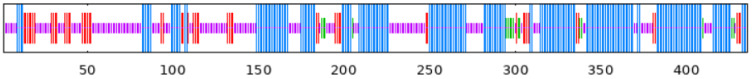 *

Blue for α-helix, purple for random coil, red for extended strand, and green for β-turn.

## Data Availability

All data generated or analyzed during this study are included in this article.

## Supplementary Materials

The following supporting information can be downloaded at: https://www.mdpi.com/article/10.3390/plants13192788/s1, Figure S1. Hydrophilic/hydrophobic analysis of all TkGGPS members; Figure S2. Transmembrane domain analysis of all TkGGPS members; Figure S3. Signal peptide analysis of all TkGGPS members; Figure S4. Multiple sequence alignment of the GGPS amino acid sequence in *T. kok-saghyz*; Figure S5. Gene expression of 7 TkGGPS genes at 0 h (control), 3 h, 6 h, 12 h, and 24 h after Ethylene and at 0 h (control), 6 h, 12 h, and 24 h after MeJA treatments in TKS leaves; Table S1. OrthoFinder result for eight species; Table S2. OrthoFinder result; Table S3. The amino acid sequence of eight species; Table S4. Collinearity relationships between TkGGPS and four species’ GGPS; Table S5. qRT-PCR primer sequence; Table S6. Gene cloning primer; Table S7. The whole proteome sequence of *Arabidopsis thaliana* (NCBI); Table S8. The whole proteome sequence of *Arabidopsis thaliana* (TAIR).
