# Supplementary material for: Genome-Wide Identification of the Geranylgeranyl Pyrophosphate Synthase (GGPS) Gene Family Associated with Natural Rubber Synthesis in Taraxacum kok-saghyz L. Rodin
Source: Plants (Basel). 2024 Oct 4;13(19):2788. doi: 10.3390/plants13192788 (PMC11478434; doi:10.3390/plants13192788)
Supplement: Supplementary file 1 [file plants-13-02788-s001.zip › Supplementary Figures.pdf]

Supplementary Figure S1. Hydrophilic/hydrophobic analysis of all TkGGPS members.

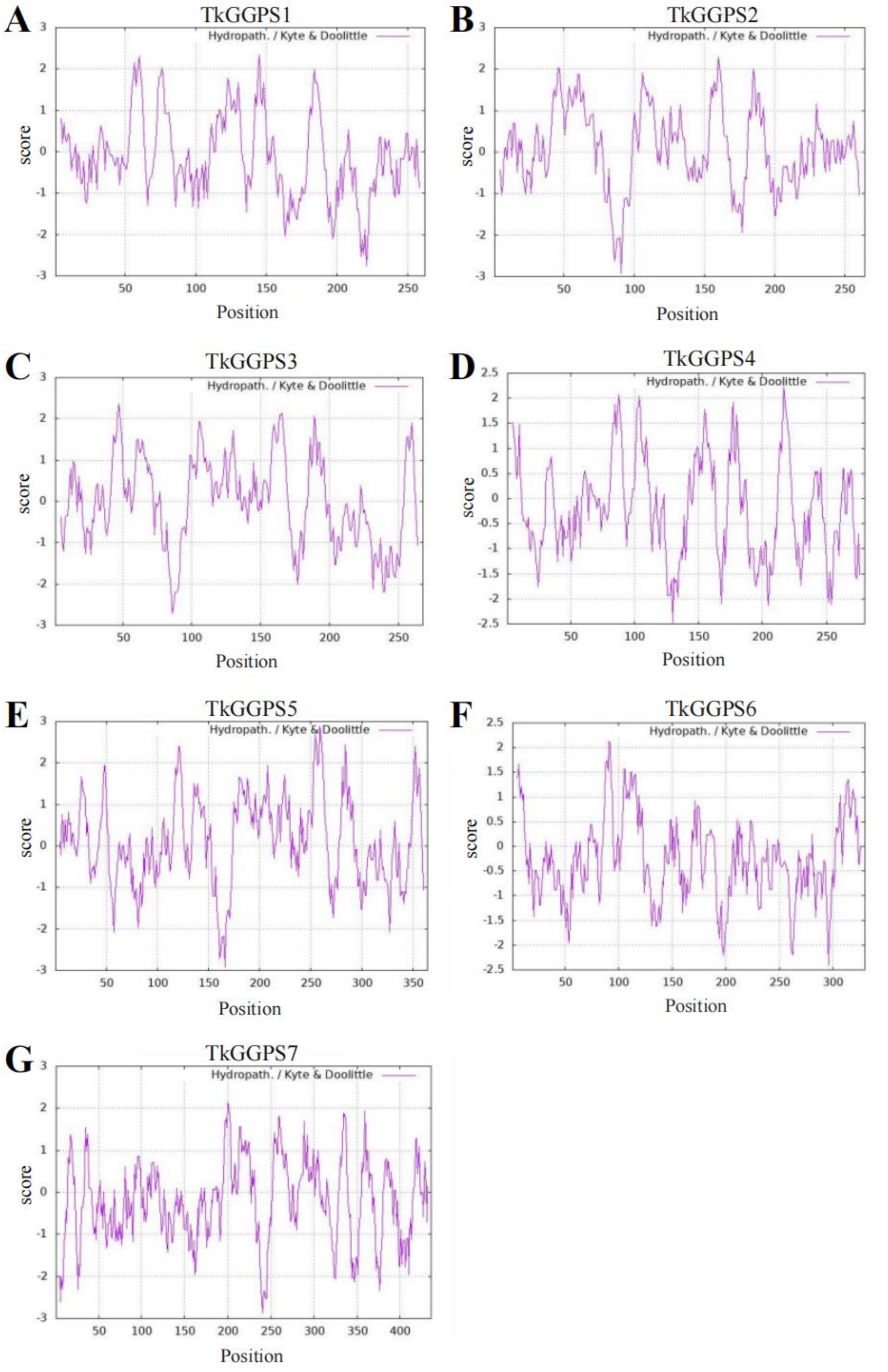

Supplementary Figure S1. Hydrophilic/hydrophobic analysis of all TkGGPS members. (A) TkGGPS1, (B) TkGGPS2, (C) TkGGPS3, (D) TkGGPS4, (E) TkGGPS5, (F) TkGGPS6, (G)

TkGGPS7. The horizontal coordinate indicates each amino acid sequence, and the vertical coordinate indicates the hydrophilic index of the amino acid. The score less than 0 indicates that the amino acid is hydrophilic, otherwise the more positive the score, the more hydrophobic the amino acid located in that region of the protein.

Supplementary Figure S2. Transmembrane domain analysis of all TkGGPS members.

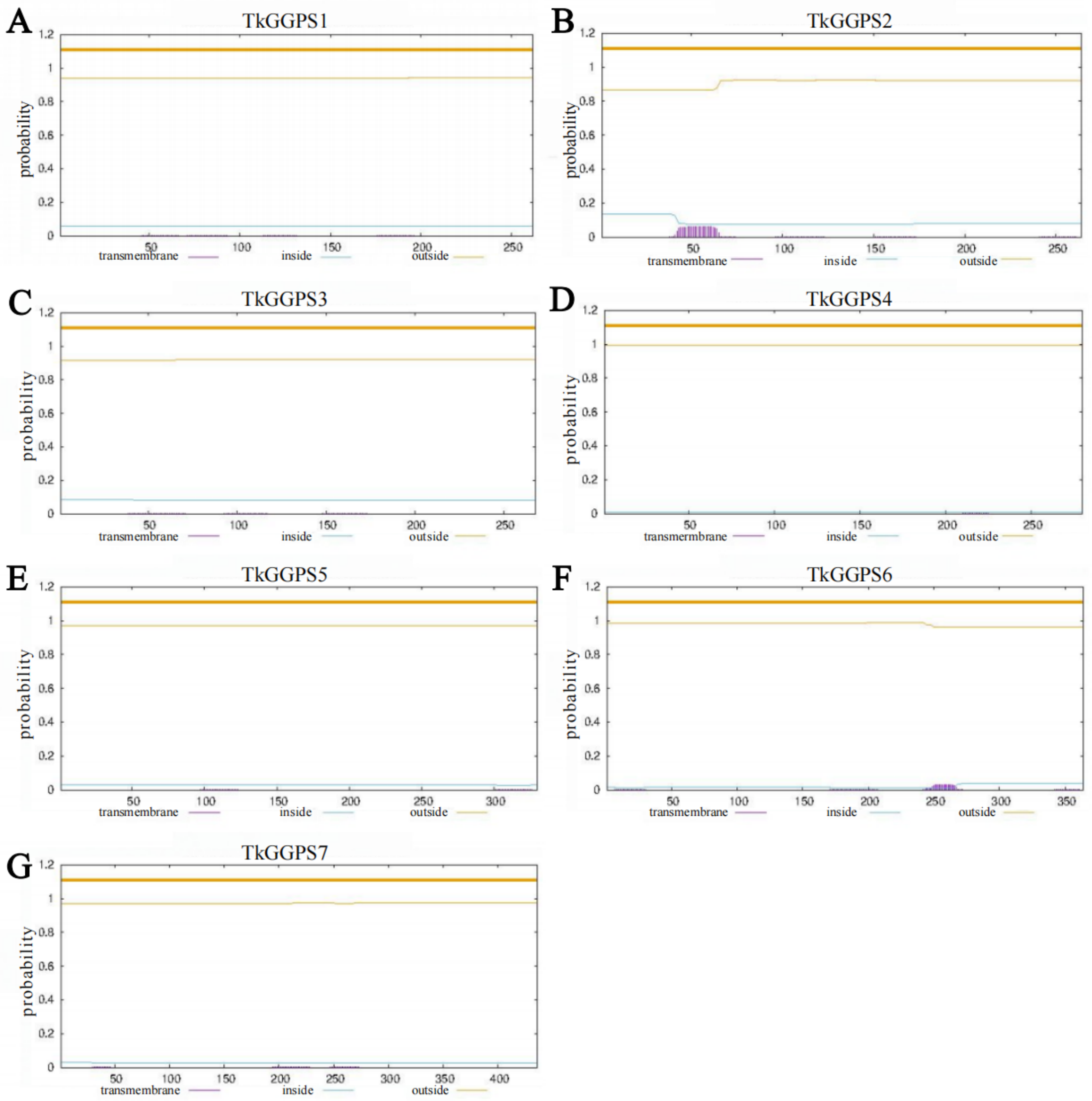

Supplementary Figure S2. Transmembrane domain analysis of all TkGGPS members. (A) TkGGPS1, (B) TkGGPS2, (C) TkGGPS3, (D) TkGGPS4, (E) TkGGPS5, (F) TkGGPS6, (G) TkGGPS7. The horizontal coordinate represents the amino acid sequence, and the vertical coordinate represents the probability of the region in which the protein sequence is located. The purple line represents the transmembrane region, the blue line represents the inside of the

membrane, and the orange line represents the outside of the membrane.

Supplementary Figure S3. Signal peptide analysis of all TkGGPS members.

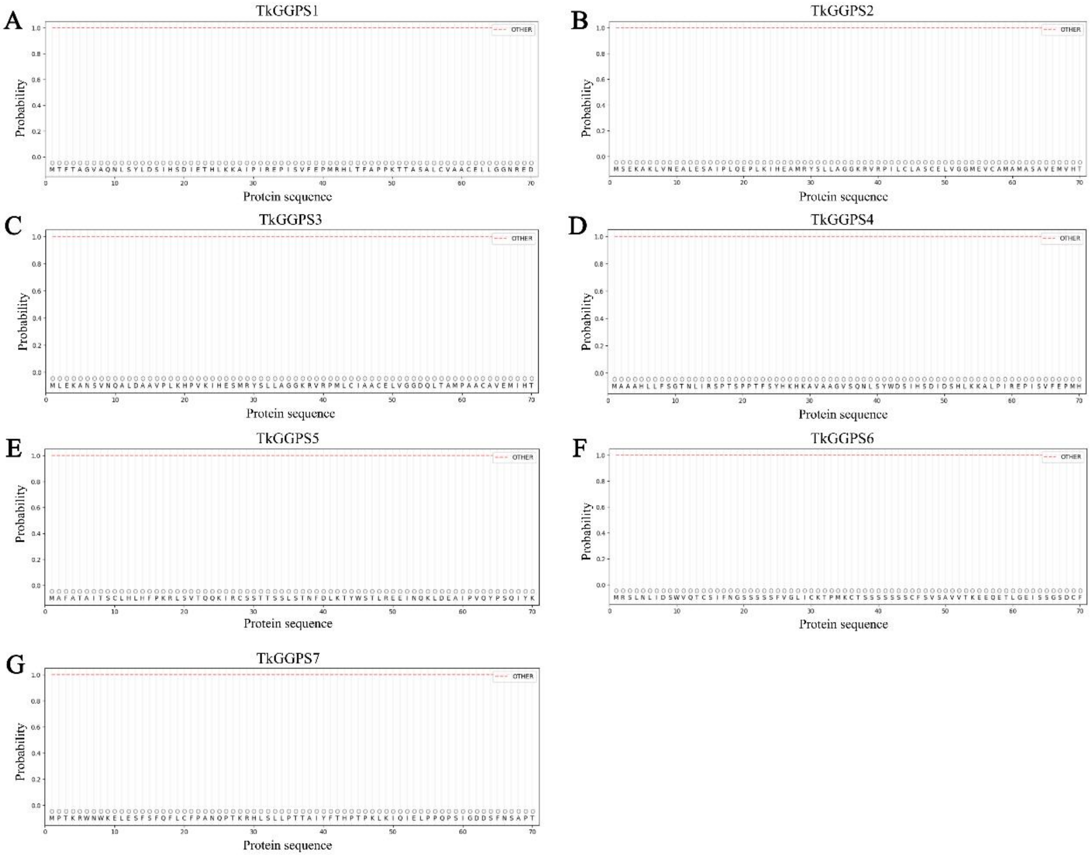

Supplementary Figure S3. Signal peptide analysis of all TkGGPS members. (A) TkGGPS1, (B) TkGGPS2, (C) TkGGPS3, (D) TkGGPS4, (E) TkGGPS5, (F) TkGGPS6, (G) TkGGPS7. The horizontal coordinate indicates the protein sequence, and the vertical coordinate indicates the presence or absence of a signal peptide.

Supplementary Figure S4. Multiple sequence alignment of the GGPS amino acid sequence in *T. kok-saghyz*.

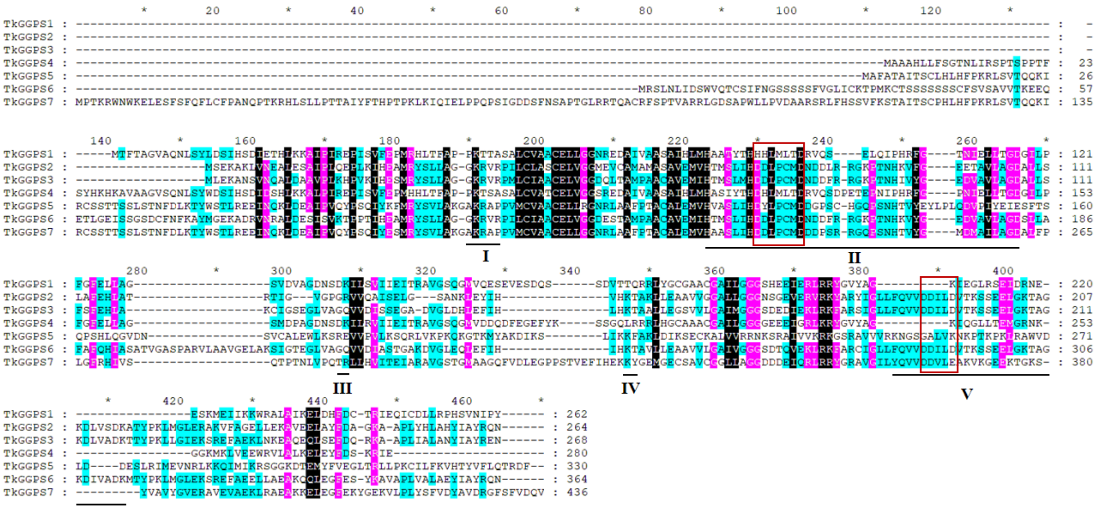

Supplementary Figure S4. Multiple sequence alignment of the GGPS amino acid sequence in *T. kok-saghyz*. There are five conservative domains, each represented by a different number and marked with a black line. The red areas are the two characteristic domains of the GGPS.

Supplementary Figure S5. Gene expression of 7 *TkGGPS* genes at 0 h (control), 3 h, 6 h, 12 h, and 24 h after Ethylene and at 0 h (control), 6 h, 12 h, and 24 h after MeJA treatments in TKS leaves.

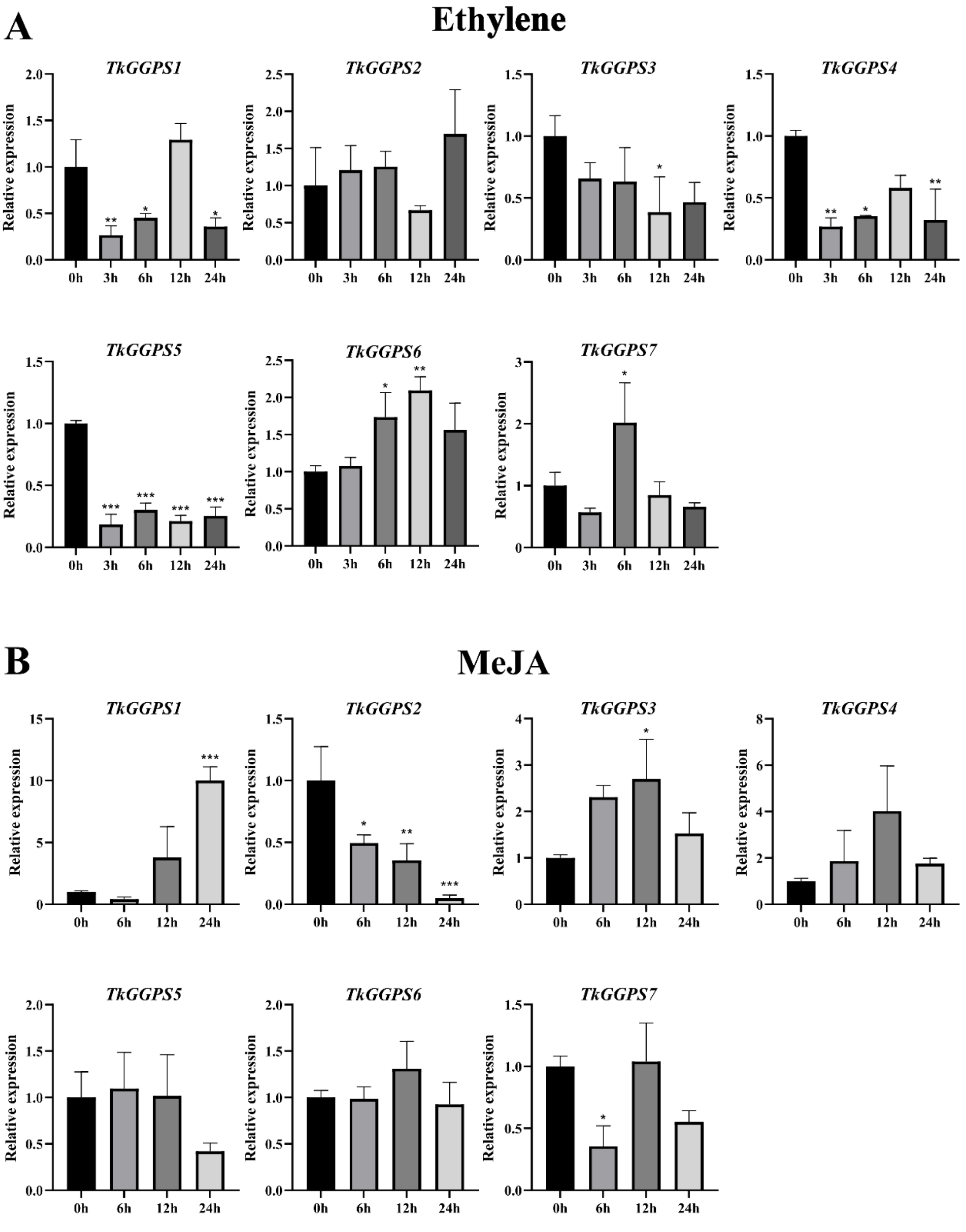

Supplementary Figure S5. Gene expression of 7 *TkGGPS* genes at 0 h (control), 3 h, 6 h, 12 h, and 24 h after Ethylene and at 0 h (control), 6 h, 12 h, and 24 h after MeJA treatments in TKS leaves. (A) Expression of *GGPS* members in TKS leaves after Ethylene treatments, (B)

Expression of *GGPS* members in TKS leaves after MeJA treatments. Data were normalized to β-actin. Vertical bars for qRT-PCR indicate the standard deviation, while an asterisk indicates the summary p-value of the independent samples t-test of the corresponding gene compared to the control (* $P < 0.05$, ** $P < 0.01$, *** $P < 0.001$).
